# Dysregulation of Liver Regeneration by Hepatitis B Virus Infection: Impact on Development of Hepatocellular Carcinoma

**DOI:** 10.3390/cancers14153566

**Published:** 2022-07-22

**Authors:** Eun-Sook Park, Mehrangiz Dezhbord, Ah Ram Lee, Bo Bae Park, Kyun-Hwan Kim

**Affiliations:** 1 Institute of Biomedical Science and Technology, School of Medicine, Konkuk University, Seoul 05029, Korea; espark97@gmail.com (E.-S.P.); bbpark1@gmail.com (B.B.P.); 2 Department of Precision Medicine, School of Medicine, Sungkyunkwan University, Suwon 16419, Korea; m.dezhbord@yonsei.ac.kr (M.D.); ahram2g@naver.com (A.R.L.)

**Keywords:** liver, liver regeneration, hepatitis B virus, HBV-related hepatocellular carcinoma

## Abstract

**Simple Summary:**

Liver regeneration after injury or partial removal is a complicated and critical process that can be affected by several factors such as hepatitis B virus (HBV) or intracellular signaling pathways. Understanding regeneration repressive mechanisms provides insight to encounter these inhibitory factors and prevent further complications including liver cancer. Here we summarized pathways of liver regeneration and pointed out in detail, the different mechanisms that may dysregulate this process during HBV infection. In addition, other intracellular factors and pathways related to the regulation of liver regeneration have been recapped. The overall information provided here can be beneficial for understanding the method of preventing and treatment of liver failure due to the defective regeneration.

**Abstract:**

The liver is unique in its ability to regenerate in response to damage. The complex process of liver regeneration consists of multiple interactive pathways. About 2 billion people worldwide have been infected with hepatitis B virus (HBV), and HBV causes 686,000 deaths each year due to its complications. Long-term infection with HBV, which causes chronic inflammation, leads to serious liver-related diseases, including cirrhosis and hepatocellular carcinoma. HBV infection has been reported to interfere with the critical mechanisms required for liver regeneration. In this review, the studies on liver tissue characteristics and liver regeneration mechanisms are summarized. Moreover, the inhibitory mechanisms of HBV infection in liver regeneration are investigated. Finally, the association between interrupted liver regeneration and hepatocarcinogenesis, which are both triggered by HBV infection, is outlined. Understanding the fundamental and complex liver regeneration process is expected to provide significant therapeutic advantages for HBV-associated hepatocellular carcinoma.

## 1. Introduction

Infection with hepatitis B virus (HBV) results in a variety of liver diseases, including acute or chronic inflammation, cirrhosis, and hepatocellular carcinoma (HCC), leading to liver injury [1,2,3].

The liver is known as the only organ that is capable of completely regenerating itself after partial hepatectomy or after being damaged by various stimuli such as toxins and pathogens. The study of liver regeneration started with a model of partial hepatectomy in rodents in 1931 [4], and research on molecular mechanisms occurring during liver regeneration has been actively conducted until recently.

The hepatocyte loss caused by long-term liver damage is compensated by activating liver regeneration signals that trigger the very complex and highly organized hepatic regeneration process which occurs through the following three steps. Many growth factors and cytokines are involved in each tightly controlled phase [5,6]. Regeneration begins when cytokines such as interleukin-6 (IL-6) are secreted from Kupffer cells in the liver. Next, hepatocyte division is activated by growth factors such as hepatocyte growth factor (HGF) and transforming growth factor-alpha (TGF-α). The final stage of regeneration is regulated by cytokines of the transforming growth factor-beta (TGF-β) family [5,6,7,8,9,10,11].

Inadequate liver regeneration causes chronic and rapid turnover of hepatocytes, which can be an important factor in liver dysfunction and eventually development of the HCC [1,2]. Balanced liver regeneration is essential for liver homeostasis. Therefore, disruption of this process by viral infection may be a major cause of various virus-mediated liver diseases, including liver cancer. In this review, we summarize the dysregulation of liver regeneration by HBV and its effect on liver regeneration in the development of HBV-related HCC. 

## 2. Pathway of Liver Regeneration

Successful liver regeneration is the restoration of the liver size and number of hepatocytes to the same level as prior to the injury. This characteristic of liver regeneration is very important for maintaining liver function homeostasis. Liver regeneration is an organized response that is induced by specific external stimuli and accompanies by successive changes in gene expression, growth factor production, and morphological structure [6].

Experimentally, the liver regeneration can be induced by various methods. Several models have been proposed for the study of liver regeneration. The most preferred method is two-thirds partial hepatectomy, which is widely used for studying liver regeneration in experimental animals [6,12]. Therefore, here, we mainly discuss the liver regeneration triggered after partial hepatectomy.

The liver regeneration process can generally be divided into three stages, namely initiation, proliferation, and termination, with various molecules participating in these three stages [7].

In the liver tissue of adults, division of hepatocytes occurs infrequent [3]. Nevertheless, following partial removal of the liver tissue, almost all the hepatocytes start to enter the replicative phase immediately [13]. Most hepatocytes are in a quiescent state and require priming before replication. Cytokines are involved in the initiation of liver regeneration. Tumor necrosis factor alpha (TNF-α) and IL-6 are the most important initiating factors in regenerating liver. After partial hepatectomy, TNF binds to the TNF receptor 1 on Kupffer cells, leading to the activation of NF-kB and production of IL-6 [14]. IL-6 activates the signal transducer and activator of transcription 3 (STAT3) and extracellular signal-related kinase 1 and 2 (ERK1/2) pathways on hepatocytes [15]. The addition of anti-TNF antibodies after partial hepatectomy significantly inhibits IL-6 production and also inhibits liver regeneration in a rat model [16]. The IL-6 knockout mouse demonstrated impaired liver regeneration with liver necrosis and failure [17]. It has been recently demonstrated that the STAT3 activity in mice is inhibited by CREB/ATF bZIP transcription factor CREBZF, which results in repression of liver regeneration. Regarding the critical role of STAT3 in promoting hepatic regeneration, controlled impeding of the CREBZF in hepatocytes may improve the liver regeneration process [18].

The epidermal growth factor (EGF) receptor ligand family and hepatocyte growth factors (HGFs) are important growth factors that are involved in the proliferation step and are responsible for cell-cycle progression [19]. HGF and its receptor-encoding gene, *c-met*, are necessary for liver regeneration and repair [20]. HGF/c-met signaling contributes to the activation of ERK1/2 [21], which induces liver cell reproduction. The EGF receptor ligand family contains EGF, TGF-α, heparin-binding EGF-like growth factor (HBEGF), and amphiregulin (AR). The Brunner’s gland is responsible for the production of the EGF, and, therefore, its extirpation decreases liver regeneration. Additionally, the EGF antiserum reduced liver regeneration significantly [22]. Following hepatocyte reproduction, the TGF-α is produced and contributes to the constant proliferation of liver cells [23]. 

The final stage of regeneration is regulated by cytokines of the TGF-β family. TGF-β suppresses cell growth and terminates liver regeneration. During the proliferation step, increased expression level of Smad7 by HGF and EGF receptors has been shown to inhibit TGF-β, which functions as an antiproliferative factor. At the termination step, however, the TGF-β production is increased from kupffer cells and upregulated stellate cells [24,25]. In addition to TGF-β, the suppressors of cytokine signaling (SOCS) block hepatocyte regeneration by downregulating the STAT3 and IL-6 signaling pathway. In another study, the mitogen-activated protein kinase kinase 4 (MKK4) function was examined in mouse models, and MKK4 was proven to work as a strong inhibitor of liver regeneration [26]. The absence of liver regeneration terminators can have detrimental consequences due to the hepatocyte hyperproliferation and liver injury.

## 3. HBV Infection and Liver Regeneration

HBV is a major pathogen that chronically infects more than 300 million people globally. Chronic HBV infection is an important cause of death through induction of cirrhosis and hepatocellular carcinoma [1,2,3]. Chronic inflammation of the liver due to the host immune response to the virus is a major contributor to the pathogenesis of HCC [27,28]. HBV X protein (HBx), a pleiotropic protein encoded by HBV, plays a crucial role in viral liver pathogenesis [29] and HBV replication [30,31,32]. HBx can also transactivate host genes by binding to many transcription factors (TFs), including AP-1 and AP-2 [33], ATF/CREB [34], C/EBP [35], NF-AT [36], p53 [37,38], HIF-1α [39], HNF1 [40], SMAD4 [41], STAT3, and NF-κB [42]. A number of studies have shown that HBx is involved in gene activation [43,44,45], apoptosis [46,47], and cell cycle control [48,49].

Studies have been conducted to understand the mechanism of regeneration by analyzing gene expression occurring in the regenerative liver. For instance, a large-scale gene expression analysis was performed to reveal the expression pattern throughout the course of liver regeneration in mice [50,51,52,53].

### 3.1. HBx Effect on Liver Regeneration by Cell Cycle Regulation

**Figure 1:**
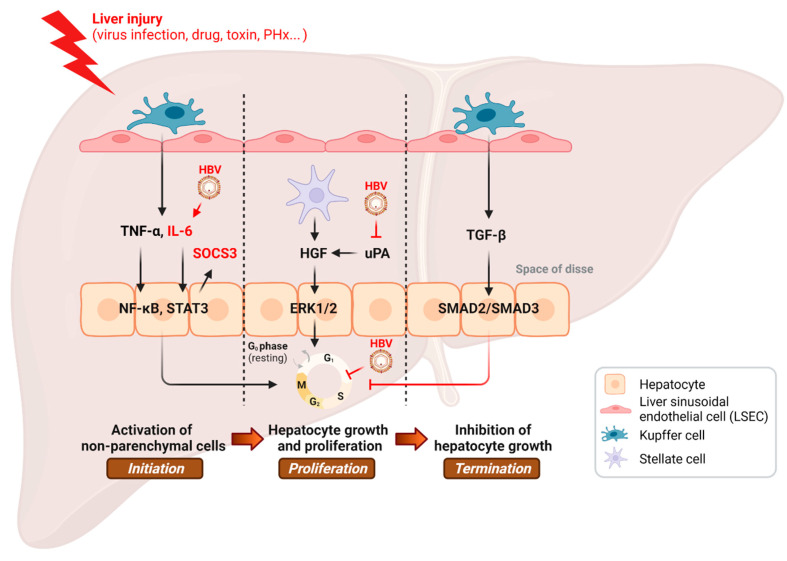
Inhibition of the hepatic regenerative pathway by HBV infection. PHx, partial hepatectomy. This illustration was created with BioRender.com.

Various studies have reported that HBV infection can impair the liver regeneration [54,55,56,57,58,59,60] (Table 1 and Figure 1). Intriguingly, HBx expression has paracrine inhibitory effect on liver cell proliferation in HBx-transgenic mice [54]. High mortality, low liver mass recovery, and impaired liver regeneration were observed in HBx-transgenic mice after partial hepatectomy. HBx protein blocks cell-cycle progression by preventing G1/S transition of the hepatocyte, and it causes both failure of liver functionality and cell death in the regenerating liver of the HBx-transgenic mice [55]. Equally important, HBx is capable of modulating significant cell-cycle regulators, such as p21 and proliferating cell nuclear antigen (PCNA), in order to disrupt normal cell-cycle checkpoints, resulting in premature entry to S phase and impeded liver regeneration [57].

### 3.2. HBx Effect on Liver Regeneration by Regulating Cellular Factors

In another investigation performed on the HBV-transgenic mice, at the initial steps of the liver regeneration, the natural killer T (NKT) cell proliferation and CD69 marker protein expression at their surface were observed. This was along with elevated levels of the interferon gamma (IFN-) production. The HBx-induced high levels of these factors were proved to be the major inhibitor of the liver generation [56].

The urokinase-type plasminogen activator (uPA) upregulates liver regeneration via activation of HGF in the extracellular matrix scaffold of the hepatocytes [61]. As a consequence, mice that are deficient in uPA or plasminogen are markedly impaired in liver regeneration [61,62]. Previously, we have reported that the HBx protein inhibits liver regeneration via downregulation of uPA expression through epigenetic regulation by way of hypermethylation of its promoter [58]. 

### 3.3. HBx Effect on Liver Regeneration by Modulating Cell Signaling Pathways

#### 3.3.1. IL-6-STAT3 Signaling Pathway

Despite what is known about the role of IL-6 in stimulating hepatocytes to grow after partial hepatectomy, the actual function of the IL-6/STAT3 pathway in tumorigenesis and liver cancer is elusive. Several studies have reported that HBx-induced IL-6 overexpression is involved in delayed liver regeneration [59]. The HBx-induced higher secretion of IL-6 was associated with an elevation of STAT3 phosphorylation, SOCS3 accumulation, and a decrease in ERK1/2 phosphorylation in the livers of HBx-transgenic mice [59]. 

#### 3.3.2. Lipid Metabolism Pathway

Cell membrane phospholipids have a critical function in lipid metabolism and liver regeneration [63]. It has been noted that elevated fat levels from adipose tissue at early stages of liver regeneration may facilitate hepatocyte proliferation. Meanwhile, it has been reported that a major regulator of glucose and lipid metabolism named hepatic pyruvate dehydrogenase kinase 4 (PDK4) level was enhanced after two-thirds partial hepatectomy [64].

The effect of HBx on the gene expression profile in the regenerated liver after partial hepatectomy of HBx-transgenic mice has been analyzed [65]. Impaired DNA synthesis and reduction in liver mass in HBx-transgenic mice have been shown to be associated with a weaker expression of genes involved in the cholesterol and nonsterol isoprenoid pathways, as well as serum amyloid A [65]. Moreover, using MALDI imaging MS analysis, we have previously reported that phosphatidylcholine biosynthesis in the regenerating liver is altered by HBV infection, causing a regeneration defect [63].

#### 3.3.3. Insulin Receptor Pathway

Finally, insulin receptor (IR)-dependent signaling represents a key factor in controlling liver regeneration [66,67]. Although HBV induces expression of the insulin receptor, liver regeneration is delayed in HBV-transgenic mice due to the intracellular holding of the insulin receptor [60]. Since the glucose is essential for cell growth, the hepatocyte proliferation rate in the liver of these mice is significantly lower than normal and is coupled with elevated fibrosis markers. 

## 4. Liver Regeneration and HCC

HCC is the most common primary liver cancer. The chronic hepatitis B virus infection, which is characterized by cycles of cell death and regeneration, is one of the most important causes of HCC globally [68]. Chronic inflammation and cytokines are key determinants in the development of fibrosis and liver cell proliferation. HBx expression has been reported to contribute to liver disease and HCC development by affecting the regeneration of the liver [57], as hepatocarcinogenesis was accelerated in HBx-transgenic mice after partial hepatectomy [69].

Although partial hepatectomy is considered as a potential treatment for HCC [70], the long-term survival of patients with HBV-associated HCC after partial hepatectomy remains challenging. The 5-year recurrence rate after surgery, which is as high as 70%, implies the low survival rate of patients [71,72,73]. The high de novo recurrence of HBV-related HCC after partial hepatectomy results from induction of the sequential changes of liver-regeneration-related SOCS family proteins, growth factors, and transcription factors, which may promote growth on the precancerous remnant liver [69]. 

In a study using liver explant from four patients with HBV-associated acute liver failure, Nissim and colleagues reported the upregulation of transcripts related to liver regeneration, including stem/progenitor markers, fibrogenesis molecules, and cell-proliferation and -tumorigenesis-related factors [74]. The authors suggested that the increased gene expression which is associated with extensive fibrogenesis is part of the repair process of damaged liver tissue, although they could not conclusively demonstrate whether the regenerative gene expression profile would have resulted in an actual successful liver regeneration due to the devastating consequences of the disease progression. However, it is important to note that, in the subpopulation of patients with submassive hepatic necrosis, hepatic stem/progenitor gene signature is accompanied with growth/proliferation and tumorigenesis-related gene expression profiles. These results denote the interesting inter-correlation among HBV pathogenesis, liver regeneration, and liver cancer. Regardless, whether non-massive chronic liver damage after HBV infection may induce dysregulated regenerative responses, along with manifestation and/or aggravation of hepatocellular cancer, remains to be determined.

### 4.1. ALR

The regulators of damage response and hepatocyte regeneration have been implicated in the manifestation and pathophysiology of HCC. For example, human augmenter of liver regeneration (ALR), a member of the ALR/Erv1 protein family with FAD-linked sulfhydryl oxidase activity catalyzing disulfide bond formation, is a hepatotropic factor and is involved in the modulation of hepatocytes’ proliferation and liver regeneration [75,76,77,78,79,80]. ALR has been shown to provide antimetastatic potential in HCC [81]. HepG2 cells expressing cytosolic 15 kDa ALR isoform (HepG2-ALR) showed reduced cell motility and epithelial-to-mesenchymal transition (EMT), as evidenced by the changes in EMT marker expression. Similarly, the decreased size of the tumor mass was observed in HepG2–ALR tumors grown in nude mice. A negative correlation of angioinvasion and tumor grading against the level of ALR, which seems to be elevated in patients with cirrhosis, cholangiocellular carcinoma (CCC), and HCC [82], has been observed in HCC patients. On the contrary, the deletion of ALR expression in liver-specific fashion exacerbates the development of HCC in mice [83]. Interestingly, ALR expression is higher in hepatoma cells and primary human hepatocytes infected with HBV compared with hepatitis C virus (HCV). Because HBV regulates the expression of ALR through activation of the nuclear factor erythroid 2 (Nrf2), it has been proposed that HBV-induced oxidative stress may be linked to the hepatic regeneration through the modulation of ALR to ensure survival of damaged cells [84]. In this regard, it is noteworthy to see that the serum ALR level of patients with HBV-infected acute-on-chronic liver failure was higher in the survival group than in the dead group, suggesting the essential role of ALR in situations where HBV induces pathological changes in liver that make it possible to use ALR as a prognosis marker in such cases [85].

### 4.2. SOCS

SOCS is a family of genes involved in the inhibition of JAK-STAT signaling pathway, which is involved in physiological and pathological regulation of cytokine and growth factor responses, as well as tumorigenesis. Among these, SOCS3 has been implicated to inhibit cytokine activation in the early stages of liver regeneration [86]. Hepatocyte-specific knockout of SOCS3 expression (*Socs3* h-KO) induced the augmentation of DNA replication and increase in liver weight after partial hepatectomy, which might be induced by the extended phosphorylation of STAT3 and activation of ERK1/2 after partial hepatectomy. The carcinogenesis was increased in *Socs3* h-KO mice, suggesting the essential role of SOCS3 as a tumor suppressor [87,88]. In HBV-infected hepatocytes, cytokine signaling pathways such as the STAT/SOCS-signaling pathway are dysregulated both in vivo and in vitro, and this is correlated with the severity of pathological inflammatory changes in the liver. It has been suggested that HBV-induced interference of STAT/SOCS signaling may serve as key molecular mechanism underlying viral pathogenesis and malignant liver tissue transformation [89]. SOCS3 hypermethylation—hence, the lower expression level of SOCS3—was significantly correlated with a poor clinical outcome of HCC patients [90]. Mechanistically, HBV induces the production of mitochondrial ROS, and the increased ROS can result in Snail-mediated epigenetic silencing of SOCS3 expression. It is proposed that the reduced expression of SOCS3 results in sustained activation of the IL-6/STAT3 pathway, which could be essential to hepatocarcinogenesis [91]. Similarly, a recent meta-analysis work demonstrated that the SOCS3 hypermethylation is associated with HBV-related HCC [92], and this may validate the possibility of SOCS3 expression or methylation status of the SOCS3 gene as a prognosis marker for HCC associated with HBV infection [93].

### 4.3. Cytokines

Several cytokines, including IL-6, TNF-α, and TGF-β, are critically involved in the modulation of liver regeneration process and carcinogenesis, as well. After partial hepatectomy, mice lacking type I TNF receptor (TNFR-1) showed massive impairment in DNA synthesis and activation of signaling pathways such as NF-κB, AP-1, and STAT3 [94]. The authors argued that the altered response is mediated by the dysregulation of IL-6 and STAT-3 signaling pathways, particularly due to the reversal of TNFR-1 KO phenotypes by the administration of IL-6 [94]. On the other hand, the excessive TNF signal has been reported to play a detrimental role in the liver regeneration processes. In Timp3 knockout mice, TNF-α converting enzyme activity was increased, thereby providing excessive release of TNF and augmentation of TNF signaling in the liver, which induced hepatitis-like inflammatory reaction and regeneration failure [95]. These phenomena are attributed to higher level of cell death induced by the increased production of TNF [95]. Recently, a concentration-dependent role for TNF-α in the modulation of hepatocyte proliferation and liver regeneration has been suggested. Zhao et al. reported that low TNF-α concentration stimulates hepatocyte proliferation and protects the liver from damage, as evidenced by the increased levels of ALT and AST in the plasma [96]. Liver damage was intensified with increasing concentrations of TNF-α, which might be regulated by a mechanosensitive transcriptional regulator, Yes-associated protein (Yap) activity. In other words, the low TNF-α concentration activated Yap1 nuclear translocation and the proliferation of hepatocytes, while a higher concentration of TNF-α inactivates Yap1, promoting hepatic cell death [96]. The increased plasma level of TNF-α and other cytokines has been reported after HBV infection, and it has been suggested that HBV (HBx) enhanced the TNF-α-induced activation of NF-κB in hepatocytes, resulting in a cooperative increase in gene expression, including those involved in the modulation of metabolic pathways [97]. Dysregulation of immune responses and hepatic proliferation may link to further damage and pathologic conversion of liver diseases from chronic hepatitis to HCC [98]. Chronic HBV infection perturbs immune responses in infected individuals [99] and may induce immune-mediated liver injury accompanied by the elevations of TNF-α and IL-1β levels [97], which also underlie HCC progression due to accelerated hepatocyte turnover rates and the accumulation of deleterious mutations [100].

### 4.4. Growth Factors

The main growth factors involved in the liver regeneration are HGF, EGF, IL-6, and TGF-β [101]. Due to the need of hepatocyte proliferation at the early stage of liver regeneration, the anti-proliferative effects of TGF-β are generally considered to be dispensable [102]. However, TGF-β plays a crucial role during the later stage by promoting EMT, inflammation, restoration of the original tissue architecture, and cell–cell interactions [103,104]. Therefore, the balance and kinetics of growth factors and TGF-β signaling during liver regeneration process should be delicately regulated [105]. Interestingly, TGF modulates uPA/PAI-1 expression, and uPA regulates TGF-β and its signaling pathway, suggesting the importance of the delicate interplay among the extracellular matrix, growth factors, uPA/PAI-1, and TGF-β. It has been suggested that TGF-β plays an essential role in the regulation of pathological consequences of HBV infection and subsequent HCC and cirrhosis of the liver [106]. This might be through the regulation of Th17 and T regulatory lymphocytes development and function by TGF-beta. Recent studies also provided evidence that TGF-β directly modulates hepatocyte function during HBV infection. Although some studies reported the anti-HBV action of TGF-β [107], it has also been suggested that HBV infection upregulated the expression of TGF-β [108,109,110,111], which might be related to the inhibition of immune responses, as well as the development of fibrosis and intra hepatic cholangiocarcinoma [110,111]. The mechanisms whereby TGF-β regulates liver pathogenesis during chronic HBV infection includes but is not restricted to the regulation of extracellular matrix system [112], regulation of immune modulators such as Th17 and regulatory T cells (Tregs) [113,114], production of cytokines and chemokines [115], regulation of miRNA signaling [116], and cell proliferation [117].

Another essential regulator of liver regeneration is HGF, a peptide with pleotropic cytokine-like functions. HGF and its target protein c-Met modulate JAK/STAT3, PI3K/Akt/NF-κB, and Ras/Raf signaling pathways in many types of epithelial cells, one of which is the liver. The GHGF/c-Met signaling pathways are critically involved in physiological processes such as cell proliferation, growth, and survival. The role of HGF in liver fibrosis, regeneration, and inflammation, as well as carcinogenesis, has been a focus for many review articles [118,119]. After partial hepatectomy, the level of HGF in the plasma increases 10 to 20 times [120], initiating the delicate process of liver regeneration ending with TGF-beta-induced rebuild up of the extracellular matrix and inhibition of HGF activity [121,122]. In HBV infected patients, the concentration of growth factors, including HGF, is increased [123,124], and this is correlated with the severity of the liver pathology [123]. In general, the increased HGF level is believed to offer survival opportunity with mechanisms involving modulation of the activity of PI3K kinase/Akt, STAT3 and cdc42-Associated Kinase 1 [124,125], as well as indirect survival promotion effects through the regulation of extracellular matrix organization [124]. Aberrant growth factor signal has been associated with the hepatocellular carcinogenesis. One of the suggested mechanisms is the overactivation of c-Met and related signals, such as the Akt pathways, resulting in increased proliferation of hepatic tissue, enhanced angiogenesis, and modulation of autophagy [126,127,128]. A recent review highlighted the importance of balance between liver regeneration signal and carcinogenesis [129]. It is reasonable to assume that HBV infection might modulate both regeneration and carcinogenesis processes through the regulation of growth factors and related signaling pathways.

One of the key factors regulating liver regeneration and carcinogenesis is extracellular matrix proteins and factors regulating the attachment and liberation of the interacting molecules, such as uPA. It has been suggested that uPA plays a crucial role in the processing and release of HGF from the hepatic extracellular matrix stores [130], and it has been suggested that the modulatory role of uPA and the plasminogen system on liver regeneration is independent of the proliferation process [131]. Signals from immune modulators and danger or pathogen sensors such as cytokines and TLRs have been implicated in the modulation of uPA expression [130]. We have previously reported that HBx can modulate the expression of uPA by regulating methylation status on its promoter region via the mechanism involving the recruitment of DNA methyltransferase (DNMT) 3A2 [58], which underlies the observed liver regeneration inhibitory effects of HBV infection. Considering the fact that uPA plays an important role in tumor invasion and progression [132], regulating uPA level and activity via HBV infection has a significant impact on the liver regeneration process and hepatocellular carcinogenesis.

### 4.5. HSPC

In the liver regeneration process, not only the mature hepatocyte proliferation, but also the cell division and differentiation of hepatic stem/progenitor cell (HSPC), plays an important role depending on the context of liver damage. In general, HSPC activation is more important in the situation where the liver loss is massive and hepatic proliferation is minimal by chronic liver diseases [133]. Interestingly, a genetic analysis of HBV-infected patients showed altered expression profiles of genes and pathways related to HSPC modulation, along with overt cell proliferation and tumorigenesis signals [74]. In a HBsAg HBV-transgenic mouse model, activation of the hepatic stem cells’ compartment was observed during hepatocarcinogenesis, suggesting the essential role of modulating HSPC activation by chronic HBV infection for the carcinogenesis [134]. The critical time window and extent of HBV infection for the modulation of mature hepatocyte proliferation and HSPC activation need to be determined further in the future and may provide insights into the correlation among HBV, liver regeneration, and carcinogenesis, as well as the means to treat the pathological outcomes.

## 5. Conclusions and Perspectives

**Figure 2:**
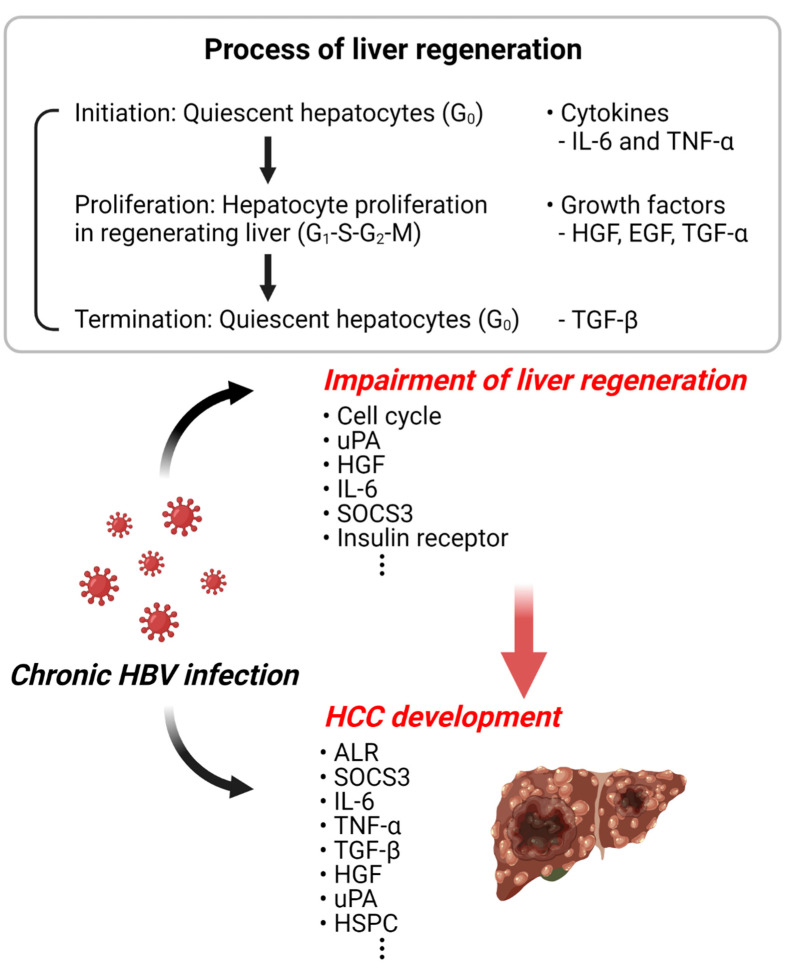
Schematic diagram of HBV-related liver regeneration and HCC. The illustration represents the increased incidence of liver cancer due to the abnormal liver regeneration caused by HBV infection. This illustration was created with BioRender.com (accessed on 18 June 2022).

HBV is one of the important and relevant risk factors for HCC. Since the liver has a strong regenerative ability, surgical resection of liver cancer or transplantation of a healthy liver is one of the treatment methods for HBV-induced liver cancer. However, the long-term survival of patients with HBV-associated HCC after partial hepatectomy is still limited. It is also not yet known how HBV infection affects liver resection or regeneration of transplanted livers. What is known is that HBV causes the disturbance of liver regeneration and promotes hepatocarcinogenesis by regulating the signal proteins involved in the liver regeneration. Although there is no direct study on the induction of liver cancer due to abnormal liver regeneration caused by HBV, the related studies strongly suggest the possibility of a relationship between the two events (Figure 2). Therefore, for the effective treatment of HBV-induced liver cancer, it is very important to study the mechanisms involved in the modulation of liver regeneration by HBV infection. A greater understanding of the regenerative process has significant benefits in treating liver failure and is expected to better explain the pathogenesis of liver cancer.

## Figures and Tables

**Table 1:** Studies on the effect of hepatitis B virus infection on liver regeneration using mouse models.

Viral Protein	Model	Injury	Effect	Mechanism	Reference
HBx	HBx-Tg mice	PHx	Inhibition of liver regeneration	Paracrine inhibitory effect	[54]
HBx	HBx-Tg mice	PHx	Failure of liver functionality and cell death	Blocking of G1/S transition	[55]
-	HBV-Tg mice	PHx	Impairment of Liver regeneration	Activated NKT cells	[56]
HBx	HBx-Tg mice	PHx	Normal liver regeneration	Premature cell cycle	[57]
HBx	Hydrodynamic Injection of viral DNA	PHx	Inhibition of liver regeneration	Epigenetic regulation of uPA	[58]
HBx	HBx-Tg mice	PHx	Delayed liver regeneration	IL6 overexpression	[59]
-	HBV-Tg mice	CCL_4_	Delayed liver regeneration	Retention of insulin receptor	[60]

-, not identified; Tg, transgenic; PHx, partial hepatectomy.
